# Glycine: a non-invasive imaging biomarker to aid magnetic resonance spectroscopy in the prediction of survival in paediatric brain tumours

**DOI:** 10.18632/oncotarget.24789

**Published:** 2018-04-10

**Authors:** Ben Babourina-Brooks, Sarah Kohe, Simrandip K. Gill, Lesley MacPherson, Martin Wilson, Nigel P. Davies, Andrew C. Peet

**Affiliations:** ^1^ School of Cancer and Genomic Sciences, University of Birmingham, Birmingham UK; ^2^ Birmingham Children’s Hospital NHS foundation Trust, Birmingham, UK; ^3^ Centre for Human Brain Health, School of Psychology, University of Birmingham, Birmingham, UK; ^4^ Medical Physics and Imaging, University Hospitals Birmingham NHS Foundation Trust, Birmingham, UK

**Keywords:** childhood brain tumours, glycine, survival, MRS, metabolism

## Abstract

Paediatric brain tumours have a high mortality rate and are the most common solid tumour of childhood. Identification of high risk patients may allow for better treatment stratification. Magnetic Resonance Spectroscopy (MRS) provides a non-invasive measure of brain tumour metabolism and quantifies metabolite survival markers to aid in the clinical management of patients. Glycine can be identified using MRS and has been recently found to be important for cancer cell proliferation in tumours making it a valuable prognostic marker. The aims of this study were to investigate glycine and its added value to MRS as a prognostic marker for paediatric brain tumours in a clinical setting.

116 children with newly diagnosed brain tumours were examined with short echo-time MRS at the Birmingham Children’s Hospital and followed up for five years. Survival analysis was performed using Cox regression on the entire metabolite basis set with focus on glycine and three other established survival markers for comparison: n-acetylaspartate, scyllo-inositol and lipids at 1.3 ppm. Multivariate Cox regression was used in conjunction with risk values to establish if glycine added prognostic power when combined to the established survival markers.

Glycine was found to be a marker of poor prognosis in the cohort (*p* < 0.05) and correlated with tumour grade (*p* < 0.01). The addition of glycine improved the prognostic power of MRS compared to using the combination of established survival markers alone.

Tumour glycine was found to improve the MRS prediction of reduced survival in paediatric brain tumours aiding the non-invasive assessment of these children.

## INTRODUCTION

Paediatric brain tumours are the most common solid tumour of childhood and the largest cause of cancer death in that patient group. Identification of high risk patients may allow for better treatment stratification and more effective disease management. Treatment may include surgical resection, radiation therapy and chemotherapy, with specific protocols based on tumour diagnosis and clinical risk factors including age at diagnosis, histopathological and molecular features and radiological imaging features [1]. Despite known risk factors, it can be difficult to identify patients in a timely manner who may respond poorly to treatment. Establishing non-invasive biomarkers of prognosis in paediatric brain tumours prior to surgery would enhance clinical management, improve disease monitoring and help to reduce treatment-related damage.

Magnetic Resonance Spectroscopy (MRS) provides a non-invasive measure of brain tumour metabolism, which can identify and quantify metabolite survival markers to aid in the clinical management of patients [2]. MRS has been previously used to identify both diagnostic and prognostic metabolite markers in adult and paediatric brain tumours [3]. Particular metabolites such as high lipids at 1.3 ppm (TLM 1.3 ppm) and scyllo-inositol (S-Ins) and glutamate (Glu) have been shown to reflect poor survival, whilst high n-acetylaspartate (NAA) and glutamine (Gln) has been shown to be a marker of good outcome in paediatric brain tumours [4, 5]. Glycine (Gly) is an additional metabolite that can be identified and quantified using MRS, and recent evidence suggests that it may be a valuable prognostic marker [5–7].

Gly is a conditionally essential amino acid, which has several physiological roles including as an inhibitory neurotransmitter in the central nervous system, cytoprotection, protein synthesis, extracellular structural support and an important role in the one-carbon metabolic cycle [8]. Notably raised in brain tumours relative to normal brain [9], elevated Gly has also been shown to distinguish high grade tumours, and has been proposed as a marker of increased malignancy in both paediatric and adult brain tumours [6, 10, 11]. Recent work also shows that an association between Gly concentration and/or related enzymes and prognosis is evident in many other tumours including breast, thyroid and colorectal cancer [12–15]. Furthermore, rapidly proliferating cells have recently been found to display an increased reliance on Gly consumption and synthesis, suggesting an important role for Gly in cancer metabolism [7]. It is therefore increasingly relevant to investigate Gly as a potential biomarker of prognosis in brain tumours and to firmly establish non-invasive ways of measuring Gly in patients.

Although histology remains the gold standard for diagnosis, not all brain tumours are surgically resected or biopsied and non-invasive MRS has proven valuable in the clinical management of these patients [16]. Metabolites measured by MRS have also been shown to provide additional information, complimentary to histology [3, 4, 17, 18]. Although prior work has shown that Gly is a potential marker of tumour grade in the brain, Gly concentration at diagnosis has not been previously associated with patient survival. Therefore, the aims of this study were to investigate Gly concentration as a prognostic marker across a cohort of paediatric brain tumours and to assess the added value it provides to the prognosis prediction potential of MRS in a clinical setting.

## RESULTS

### Patients

The brain tumour cohort consisted of 98 patients after quality control criteria were applied, with 18 removed during the quality control process. The patient cohort consisted of 81 biopsied tumours and 17 unbiopsied tumours. There were 59 survivors (60%) and 39 non-survivors by the end of the 5 year follow up period. All patient deaths were as a result of their disease. The mean age (±SD) of the patient cohort at diagnosis was 7.51 (±5.4) years, and 71% of patients were male. Age and gender were not found to be predictive of survival in this cohort with log-rank values of 1.2 and 0, respectively. The patient tumour type distribution with associated frequency and deaths within the 5 years is shown in Table 1. The tumour type survival rates in the cohort were consistent with prognosis expectations for the more common tumour types.

**Table 1:** A summary of patient tumour type diagnoses in the cohort, based on clinical information, imaging and histopathology where available, patient deaths, mean metabolite (Gly, NAA, TLM 1.3 ppm and S-Ins ) concentrations and mean relative risk for the groups (where *n* > 2)

Tumour Type	Patients (*n*)	Deaths (*n*)	Gly (SD), mM	NAA (SD), mM	TLM 1.3 ppm (SD), mM	S-Ins (SD), mM	MRS Relative Risk
Pilocytic Astrocytoma	23	1	0.58 (1.13)	0.74 (0.78)	8.35 (6.47)	0.01 (0.06)	0.74
Unbiopsied Optic Pathway Glioma	6	0	0 (0)	1.34 (1.01)	2.24 (2.77)	0.10 (0.23)	0.50
Ependymoma	5	3	3.49 (5.98)	0.25 (0.18)	14.21 (9.66)	0.29 (0.32)	1.29
Diffuse Astrocytoma	4	2	0.34 (0.56)	0.69 (0.72)	9.55 (10.45)	0.10 (0.18)	0.82
Diffuse Intrinsic Pontine Glioma	9	7	0.56 (0.84)	1.59 (1.63)	5.08 (6.58)	0.31 (0.27)	0.92
Atypical Teratoid Rhabdoid Tumour	3	3	0.94 (0.91)	0.14 (0.12)	33.73 (22.14)	0.11 (0.18)	2.16
Medulloblastoma	21	13	3.57 (2.78)	0.23 (0.25)	21.05 (17.34)	0.37 (0.37)	1.96
Tectal Plate Glioma	4	0	0.02 (0.05)	1.26 (1.06)	1.83 (1.66)	0.06 (0.08)	0.64
Glioblastoma	5	5	0.66 (0.81)	0.42 (0.19)	16.93 (8.99)	0.18 (0.13)	1.43
Germinoma	3	0	0.62 (0.52)	0.48 (0.64)	11.28 (7.76)	0.14 (0.18)	1.47
High Grade (III & IV)	38	23	2.00 (2.52)	0.46 (0.61)	18.29 (17.30)	0.24 (0.31)	1.74
Low Grade (I & II)	49	15	0.53 (0.97)	0.92 (1.01)	7.06 (6.79)	0.11 (0.21)	0.65
Ungraded	11	1	0.56 (0.90)	0.57 (0.60)	15.95 (11.80)	0.19 (0.34)	1.15

The cohort was also split into ungraded (*n* = 11), High and low WHO grade groups (*n* = 87) for metabolite concentration and relative risk comparison. Rarer tumour types, where *n* < 2, were included in the high grade, low grade and ungraded groups and also included in the survival analysis.

### Glycine is a marker of poor prognosis

**Figure 1:**
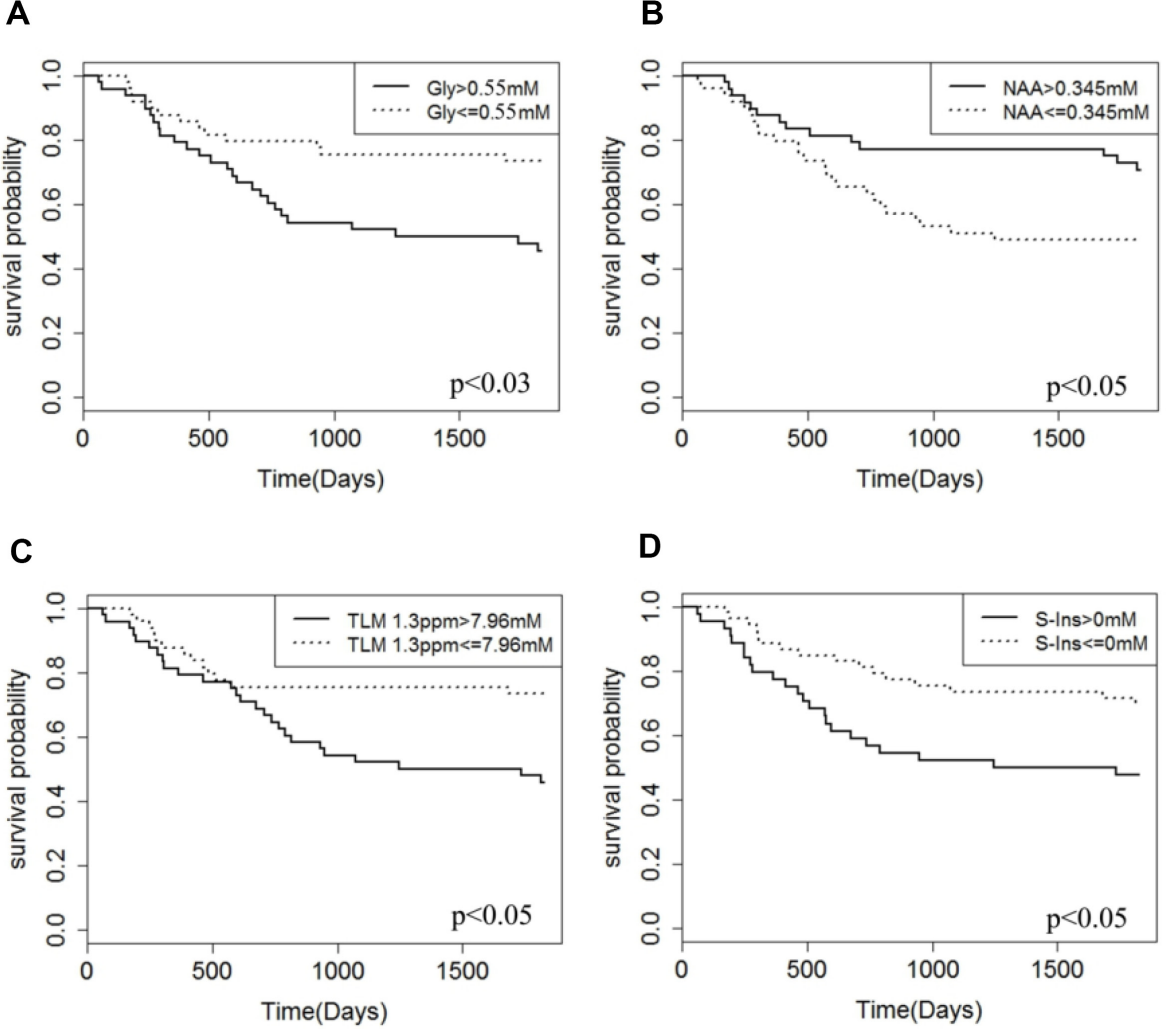
Kaplan Meier 5-year survival curves for the patient cohort Gly (**A**), NAA (**B**), TLM 1.3ppm (**C**) and S-Ins (**D**) are presented. High & low concentration groups are based on optimised cut-off values.

**Figure 2:**
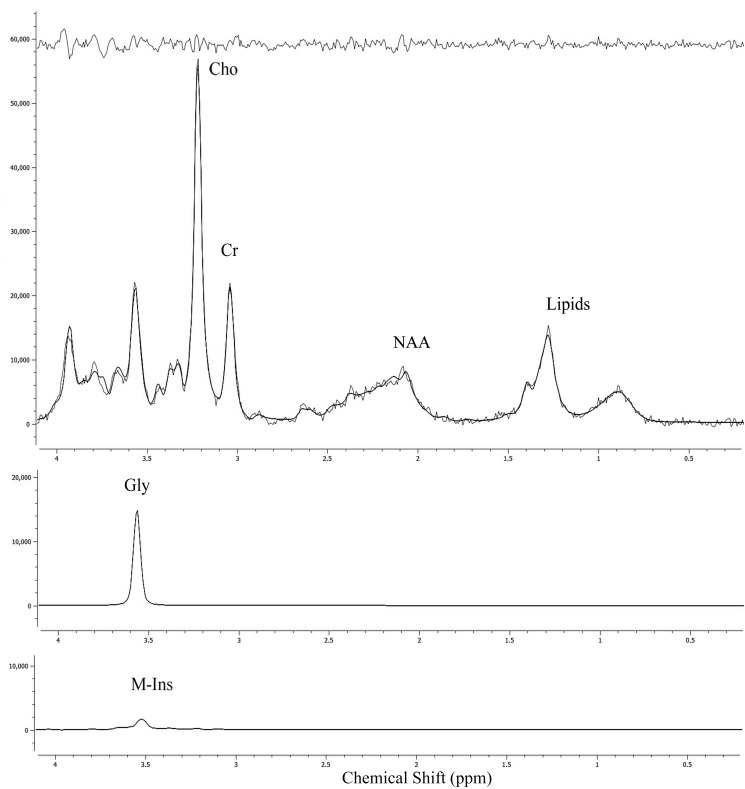
An example medulloblastoma patient MRS spectrum with TARQUIN peak fits of Gly, M-Ins and fit residuals shown The medulloblastoma spectrum shows high Gly, low M-Ins and this patient died within the 5 year follow-up period. Examples of an ependymoma and pilocytic astrocytoma spectrum are additionally shown in Supplementary Materials.

Gly was found to be a predictor of poor prognosis (*p* < 0.03) in the patient cohort based on a univariate Cox regression analysis and a concentration cutoff of 0.55 mM (Figure 1, Table 2). Gly concentrations were found to be significantly higher the high grade tumour group (2.00 ± 2.52 mM) compared with the low grade tumour group (0.53 ± 0.97 mM; *p* < 0.01) (Table 1) and concentrations correlated with WHO grade of the tumours, (*r* = 0.44; *p* < 0.01). Example spectra from a medulloblastoma patient case highlight high Gly levels with the associated poor prognosis of the patient (Figure 2). Additional spectra of an ependymoma and pilocytic astrocytoma patient (Supplementary Figures 1 and 2) highlight that low Gly concentration also predicted the survival of the patient correctly in those cases. Gly was also found to be a significant survival marker after the medulloblastoma group was removed from the analysis (*p* < 0.05).

**Table 2:** A summary of univariate Cox regression survival hazard ratios, log-rank test and significance values results for the individual metabolite survival markers Gly, NAA, TLM 1.3 ppm and S-Ins

	Gly	TLM 1.3 ppm	S-Ins	NAA
**Hazard ratio**	2.29	2.30	2.14	0.49
**Range: low-upper**	1.17–4.45	1.14–4.32	1.13–4.05	0.25-0.94
**Log-rank**	6.30	4.70	6.50	5.80
**Likelihood *p*-value**	0.008	0.01	0.02	0.03

To validate the Gly concentrations a comparison between matched short and long echo-time MRS was conducted and a strong correlation was found (*r* = 0.91; *p* < 0.001). An example long echo spectrum and a graph of the Gly concentration correlation between echo times is shown in Supplementary Figures 3 and 4.

### Established survival marker comparisons

Established survival metabolite markers TLM 1.3 ppm and S-Ins were also found to be predictors of poor prognosis and NAA was found to be a predictor of good prognosis (Figure 1, Table 2). Hazard ratios and log-rank tests showed that the prognosis prediction potential of Gly was similar to other individual metabolite markers (Table 2).

### The addition of glycine improves the MRS prediction of prognosis

**Figure 3:**
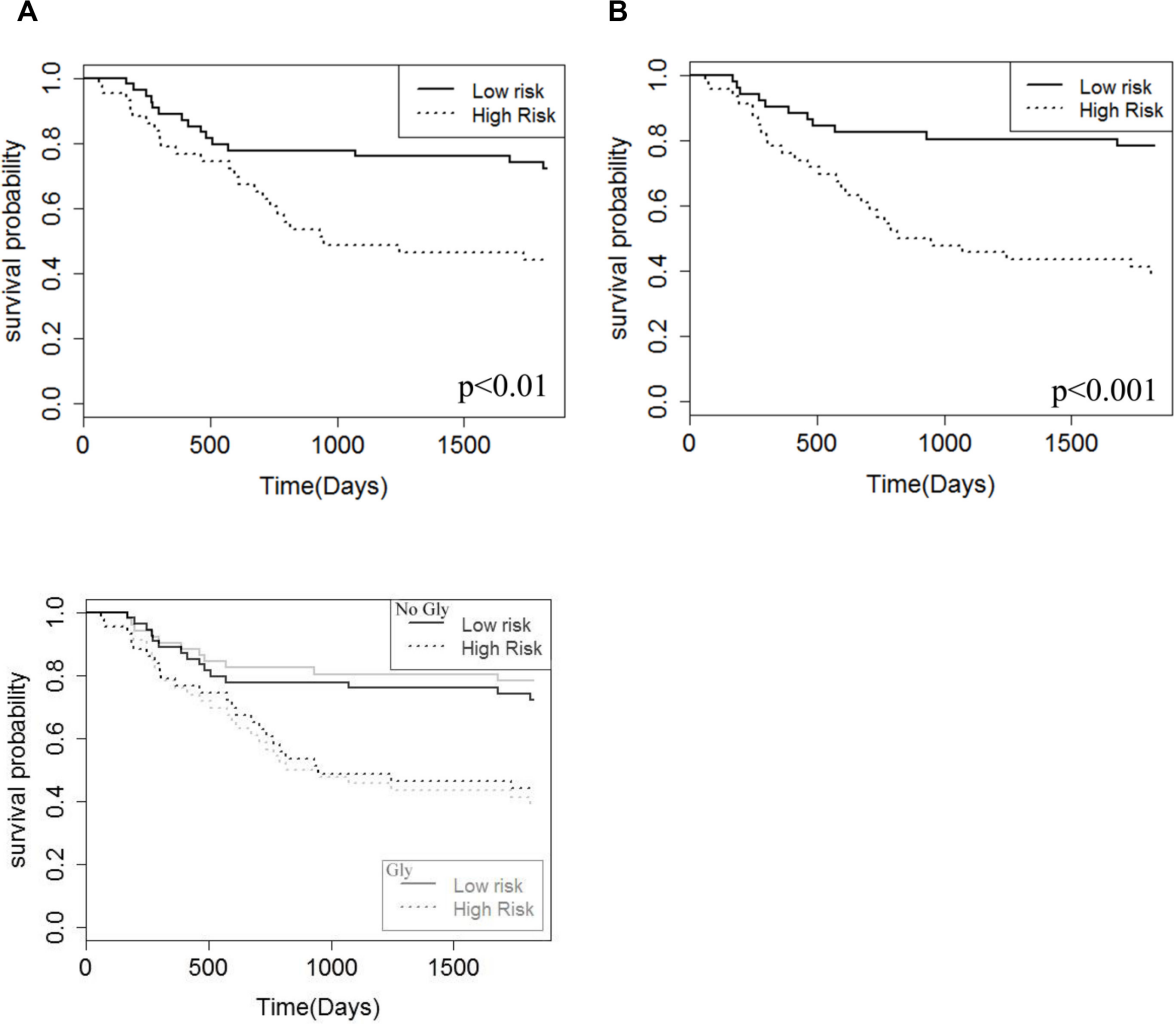
Kaplan–Meier survival curves for the patient cohort based on a risk analysis and multivariate Cox regression of the four metabolite survival markers, Gly, NAA, S-Ins, TLM 1.3 ppm The two Kaplan–Meier curves show the analysis without Gly (**A**) and with Gly included (**B**) in the analysis. Median cutoffs were used to define the low and high-risk groups.

A multivariate Cox regression was used to combine metabolite markers in the analysis. The log-rank test results of 14.4 and 7.7 for the analysis with and without Gly, respectively, show the survival prediction was significantly higher with Gly in the analysis (*p* < 0.05). This result was also seen in the Kaplan Meier curves based on risk values, where larger separation of the high risk and low risk groups was seen in the analysis with Gly included (Figure 3).

### MRS prediction of survival in individual tumour types

Mean MRS relative risk for tumour types with *n* > 2 were reported in Table 1. The MRS relative risk value predicted tumour types of good prognosis in this cohort, pilocytic astrocytoma, unbiopsied optic pathway glioma and tectal plate glioma all had mean values of less than 1. Poor prognosis tumour types in this cohort, medulloblastoma, atypical teratoid rhabdoid tumour, glioblastoma and ependymoma, were also identified well through MRS relative risk with mean values greater than 1. However, prognosis for Diffuse Intrinsic Pontine Glioma *(DIPG)*, diffuse astrocytoma and germinoma tumour types was not well predicted through relative risk alone. Observing individual results, MRS relative risk in these tumour types only predicted the correct prognosis in 2 out of 9 DIPG patients, 2 out of 4 diffuse astrocytoma and 1 out of 3 germinoma patients. The high grade tumour group had a high MRS relative risk and low grade tumour group had a low MRS relative risk

## DISCUSSION

This study has established that tumour Gly concentration, measured non-invasively by MRS, is a prognostic marker in a cohort of paediatric brain tumour patients. A higher concentration of Gly was significantly associated with reduced survival, indicating that Gly is a marker of poor prognosis across a wide range of paediatric brain tumours. Furthermore, the addition of Gly to the existing set of metabolites previously associated with survival in brain tumours improved the overall prognosis prediction potential of MRS. We also detected a significant correlation between tumour grade and Gly concentration in the smaller cohort of cases where the tumour was biopsied. Although these results agree with prior work of Gly concentration as a marker of malignancy [6, 10, 11, 19–23], this is the first study to formally show that Gly is a predictor of survival and not just grade in brain tumours.

Recent evidence suggests that Gly has a particularly important role in cancer metabolism perhaps explaining why Gly concentration is higher in more aggressive tumours. Gly is particularly linked to the one-carbon metabolic cycle whereby non-essential amino acids donate carbon units to produce complex metabolic reactions within folate, methionine and transsulfuration pathways including regulation of cell biosynthesis, redox status and epigenetic status [8, 24, 25]. Gly metabolism and the one-carbon metabolic cycle has been directly linked to increased cellular proliferation via the Gly cleavage system in several cancers including non-small cell lung cancer, breast and thyroid cancer [14, 15, 26]. This is evidenced by the upregulated activity of Gly metabolic enzyme Gly decarboxylase (GLDc) and an association between poor prognosis subgroups and increased GLDc staining using immunohistochemistry in these tumours [14, 15, 27]. Indeed the gene that underlies GLDc is now being proposed as a metabolic oncogene, and novel therapies specifically targeting Gly metabolism via the one-carbon cycle are being proposed to disrupt cellular proliferation and prevent tumourigenesis [26]. Although there is no evidence to date of increased GLDc activity in brain tumours, it seems likely that Gly metabolic activity may be upregulated, particularly in high grade brain tumours. An association between increased tissue Gly concentration and progression free survival in *ex vivo* colorectal cancer tissue has been the only study to date to link Gly concentration measured by MRS with adverse prognostic outcome [13].

The detection of Gly by MRS is potentially hindered owing to the spectral overlap with Myo-Insositol ( M-Ins). However, literature reports provide evidence that Gly in tumours can be reliably detected using the methods applied in this study. [6, 28]. M-Ins levels are known to decrease more rapidly than Gly levels with echo-time, hence the Gly peak fit in long echo-time MRS is more robust. The strong correlation between the matching short and long echo-time Gly concentration found in this study (Supplementary Figure 4) provides confidence that Gly in the short echo-time MRS has been fitted well. Additionally, M-Ins has a peak at 4.06 ppm, which can be used to aid distinction between Gly and M-Ins if a large contribution of M-Ins is present in the 3.56 ppm peak. This peak is modelled and fitted by TARQUIN as part of the quantitative analysis and may contribute to the ability of the method to distinguish Gly and M-Ins in this study. Other groups have reported that Gly concentrations measured in brain tumours by *in vivo* short echo-time MRS at 1.5T correlate with Gly levels determined from *ex vivo* tissue samples and other *in vivo* methods [6, 28]. The concentrations of Gly reported in this study (Table 1) are comparable to the reported literature values for low grade and medulloblastoma tumours [6]. Medulloblastoma tumours have higher Gly levels compared with other tumours and relatively poor prognosis. However, removing these tumours from the analysis we still found Gly to be a marker of overall survival, showing that Gly was not purely a marker of this tumour type. Gly concentrations correlated with grade in this study, which is in agreement with reported literature studies and supports the measure as a survival marker for brain tumours [6, 7, 11, 28].

It was also important to establish the added value of Gly to the overall survival prediction of metabolite markers measured by MRS. We found that NAA, S-Ins and TLM 1.3 ppm were individual markers of prognosis in this cohort, which is consistent with prior results reported in paediatric brain tumours [4, 29]. Glutamine has previously been reported as a marker of good prognosis but was not found to be so in this study [4]. Glutamine is poorly determined by MRS at 1.5T and further study of this metabolite at higher field strength are warranted. NAA is regarded as a marker of neuronal activity, and while its role in tumours is not well understood, higher concentrations of NAA detected in brain tumours is associated with better outcome [29]. Using the current methodology, the peak around 2 ppm is largely fitted to the NAA spectrum consistent with many previous studies. However, important contributions to this peak may arise from other molecules and the biological interpretation of the prognostic significance of NAA should be undertaken with caution [30]. Additionally, contributions to the NAA peak can occur from non-tumour tissue if the voxel is close to the tumour edge. However, review of the cases with high NAA values showed that the majority of cases with high NAA were diffuse tumours with voxels generally well within the radiological abnormality.

S-Ins has been previously associated with poor prognosis in paediatric brain tumours, however, its role in tumour metabolism is not well known, warranting further investigation [4, 31]. Lipids are well documented as being associated with poor prognosis in both paediatric and adult brain tumour patients being associated with apoptosis, necrosis and cytoplasmic lipid droplets [32–35]. The results showed Gly complimented the established survival markers and increased prognostic value. Therefore, the addition of the Gly measure in MRS provides increased power of the technique as a prognostic tool [4].

There is clinical interest in establishing non-invasive markers of prognosis in individual tumour types and the prediction of individual outcome. In this study this was preliminarily investigated through the MRS derived relative risk value. The high grade grouping of tumours had a significantly greater relative risk value than the low grade tumour group. Looking at more specific tumour types, the relative risk value was good at predicting prognosis for most tumour types, but performed the best for groups that consisted of high numbers of patients with either good or poor survival, e.g. pilocytic astrocytoma and atypical teratoid rhabdoid tumours. The exceptions to this provide an interesting insight into the information provided by MRS. Diffuse astrocytoma and DIPG tumour groups progress to a higher grade prior to death and may evolve to a more aggressive MRS profile during tumour progression [36]. Germinomas are very rapidly growing tumours which metastasise readily, consistent with their MRS profile, but are extremely sensitive to treatment. MRS profiles therefore appear to provide information on inherent tumour properties of growth and malignant potential rather than response to treatment. In future work, larger cohorts should investigate specific markers of survival for individual tumour types and the ability of MRS to predict response to specific treatments.

Non-invasive measures of diagnosis and prognosis in brain tumours are becoming increasingly important. This is particularly evident given that approximately a quarter of paediatric brain tumours are not biopsied and therefore receive no histological diagnosis. In this instance, metabolites can provide additional clinical information non-invasively. It is important to note that MRS has been successfully used to discriminate relapse from treatment related effects, to non-invasively identify tumour type and to provide additional molecular genetic information to aid in clinical management [4, 17, 18, 37, 38]. However, it can remain difficult to identify patients at diagnosis who respond poorly to treatment, despite known molecular and histopathological risk factors. Metabolite prognostic markers show potential for providing additional information to aid in clinical decision making prior to surgery. Hence the current work showing Gly, in combination with established MRS survival markers, as a prognostic marker across a range of brain tumours shows promise for implementation into clinical practice, and may prove useful for risk stratification and enhanced disease management.

In conclusion, Gly was found to be a marker of poor overall survival using non-invasive MRS in paediatric brain tumours. This is an important finding as Gly has been recently found to be vital for cancer cell proliferation in tumours. The addition of Gly to other reported metabolite survival markers improved the prognosis prediction potential of MRS, thus, improving MRS as a tool for aiding in the clinical management of brain tumour patients.

## MATERIALS AND METHODS

### Patients

116 children with newly diagnosed brain tumours were examined with MRS prior to treatment as part of their clinical investigations at the Birmingham Children’s Hospital. The accrual period was between September 2003 and July 2009, with all patients followed up for five years. The cohort overlaps with that from a previously reported study and so should not be considered a an independent validation of the biomarkers reported in that study [4]. Rather it is an investigation of the value of glycine as a biomarker and its added value compared with other metabolite biomarkers. Clinical data was obtained through the West Midlands Tumour Registry and clinical records, which included gender, patient age at diagnosis and dates of death. Tumour diagnosis was confirmed by a multidisciplinary team using histopathology, along with clinical and radiological information. All graded tumours were biopsy proven. Ungraded tumours were either unbiopsied or biopsied and found to have a WHO diagnosis with no associated grade. Patients were given standard treatment based on their diagnosis, tumour stage, age and extent of resection. Treatment included a variety of methods including surgical resection, radiotherapy and chemotherapy according to their clinical need and not based on Gly levels. Study approval was gained through research ethics committee and signed consent given by parents or guardians. The study was designed following the REMARK criteria [39].

### MRI – protocols and analysis

MRI and MRS were acquired pre-treatment on a 1.5T Siemens Symphony Magnetom with a single-channel head coil and 1.5T GE Signa Excite scanner with an 8-channel head coil. MRI included standard T1- and T2-weighted images of the brain, diffusion-weighted imaging followed by gadolinium contrast administration and then further T1-weighted imaging. MRI was utilised to provide anatomical reference to the primary tumour and measure contrast enhancement within the tumour. The MRS voxel was placed within the solid component of the primary tumour measuring 1.5 or 2 cm sided cube depending on the size of the tumour. PRESS (Point Resolved Single Voxel Spectroscopy) localisation was used with a repetition time of 1500 ms and an echo-time of 30 ms. Fifteen patients had matching long echo-time (135 ms) MRS data, which was analysed as a validation method for the Gly fit. Water suppressed data were acquired with 256 or 126 repetitions, depending on voxel size for signal averaging. Water unsuppressed data were additionally taken as a metabolite concentration reference. MRS data were transferred from the scanner to a PUKKAJ PACs system (version 6.6.1) used for storage of research and advanced MRI data.

Unprocessed MRS signals were analysed using the TARQUIN [40] software package (version 4.3.6). Each spectrum was fitted with the following metabolites in the basis set; Alanine (Ala), Aspartate (Asp), Citrate (Cit), Creatine (Cr), γ-aminobutyric acid (GABA), Glycero-PhosphoCholine (GPC), Glucose (Glc), Glutamine (Gln), Glutathione (Glth), Glutamate (Glu), Glycine (Gly), Myo-Inositol (M-Ins), Lactate (Lac), Lipids (0.9 ppm, 1.3 ppm, 2.0 ppm), Macromolecules (0.9 ppm, 1.2 ppm, 1.4 ppm, 1.7 ppm, 2.0 ppm), N-AcetylAspartate (NAA), N-AcetylaspartylGlutamate (NAAG), PhosphoCholine (PCh), PhosphoCreatine (PCr), Scyllo-Inositol (S-Ins) and Taurine (Tau). No restrictions were placed on relative intensities of the lipid and macromolecule peaks fitted. For statistical tests the following metabolite pairs were combined as they are known to be difficult to resolve reliably: NAA and NAAG (TNAA), PCh and GPC (TCho), PCr and Cr (TCr) and finally lipids and macromolecules to TLM 0.9 ppm, TLM 1.3 ppm, TLM 2.0 ppm. Metabolite concentrations were scaled relative to the water signal with a water molarity assumed to be 35880 mM [40].

Individual spectra were inspected by two expert spectroscopists blinded to the survival data and excluded if they did not meet one or more of the quality control criteria. These were displaced voxel location (too close to lipid containing structures), unstable spectral baseline, presence of spectral artifacts, signal-to-noise ratio (SNR) <5, overall metabolite linewidths >0.15 ppm and water linewidth >10 Hz. Voxels containing more than 10% of uninvolved brain as assessed by the conventional MRI were excluded from the analysis.

### Statistical analysis

All metabolites in the basis set were utilised to test whether they were markers of prognosis through a univariate Cox regression, log-rank test and Kaplan–Meier analysis using a median metabolite concentration cutoff. Metabolites that were found to be significant (*p* < 0.05) had their metabolite concentration cutoff optimised iteratively based on the lowest *p*-value. Gly and established metabolite survival markers, TLM 1.3 ppm, NAA and S-Ins were additionally optimised in the same manner. Metabolite concentrations for all tumours were calculated, and mean values for each tumour diagnostic group where *n* > 2 are shown in Table 1. Metabolite concentrations of rare tumour types, where *n* < 2, were not shown in Table 1 but were included in the survival analysis. These included 1 anaplastic ependymoma, 2 astrocytomas, 1 ganglioglioma, 1 mixed cell germ tumour, 2 choroid plexus papilloma, 1 pineoblastoma, 1 secreting germ cell tumour and 1 biopsied metastatic tumour. The Gly concentration is known to be high in medulloblastoma tumour therefore a univariate Cox regression analysis of Gly concentration was performed with these tumours removed to assess any potential bias in survival from this group. All tumours with associated WHO grades were split into low grade (grade I and II) and high grade (grade III and IV) groups with the remainder placed into the ungraded group to compare mean Gly concentration values (Table 1). A Spearman’s correlation test was also performed establish if WHO grade correlated with Gly concentration. Additionally, Gly concentrations from the long and short echo-time MRS in the same scan session in patients where available were tested for correlation to validate the short echo-time Gly fits.

MRS provides multiple markers of tumour prognosis, hence while establishing new individual markers is of clinical interest the combination of markers may provide more predicting power. Therefore, the added value of Gly to the survival prediction of MRS was assessed by combining established survival markers with Gly included or excluded from a multivariate Cox regression analysis. Risk prediction analysis, using the multivariate Cox regression model, was performed to assess the MRS prognosis prediction, with and without Gly included. This was visualised through Kaplan–Meier survival curves using median risk score cutoffs.

Risk analysis, was also performed to assess the ability of MRS as a whole to predict individual tumour type prognosis (Table 1). Survival analyses were performed in R statistical software using the survival library. A *p*-value of < 0.05 was deemed significant in all tests.

## SUPPLEMENTARY MATERIALS FIGURES



## Abbreviations

Ala: Alanine
Asp: Aspartate
Cit: Citrate
Cr: Creatine
DIPG: Diffuse Intrinsic Pontine Glioma
GABA: γ-aminobutyric acid
GPC: Glycero-PhosphoCholine
Glc: Glucose
Gln: Glutamine
Glth: Glutathione
Glu: Glutamate
Gly: Glycine
GLDc: Gly decarboxylase
MRS: Magnetic Resonance Spectroscopy
M-Ins: Myo-Inositol
Lac: Lactate
MM: Macromolecules
NAA: N-AcetylAspartate
NAAG: N-AcetylaspartylGlutamate
PCh: PhosphoCholine
PCr: PhosphoCreatine
PRESS: Point Resolved Single Voxel Spectroscopy
S-Ins: Scyllo-Inositol
SNR: Signal-to-Noise Ratio
Tau: Taurine
TNAA: Total N-AcetylAspartate
TCho: Total Choline
TCr: Total Creatine
TLM: Total Lipids and Macro molecules
WHO: World Health Organisation
