# Supplementary material for: Glycine: a non-invasive imaging biomarker to aid magnetic resonance spectroscopy in the prediction of survival in paediatric brain tumours
Source: Oncotarget. 2018 Apr 10;9(27):18858–68. doi: 10.18632/oncotarget.24789 (PMC5922361; doi:10.18632/oncotarget.24789)
Supplement: Supplementary file 1 [file oncotarget-09-18858-s001.pdf]

Glycine: a non-invasive imaging biomarker to aid magnetic resonance spectroscopy in the prediction of survival in paediatric brain tumours

SUPPLEMENTARY MATERIALS

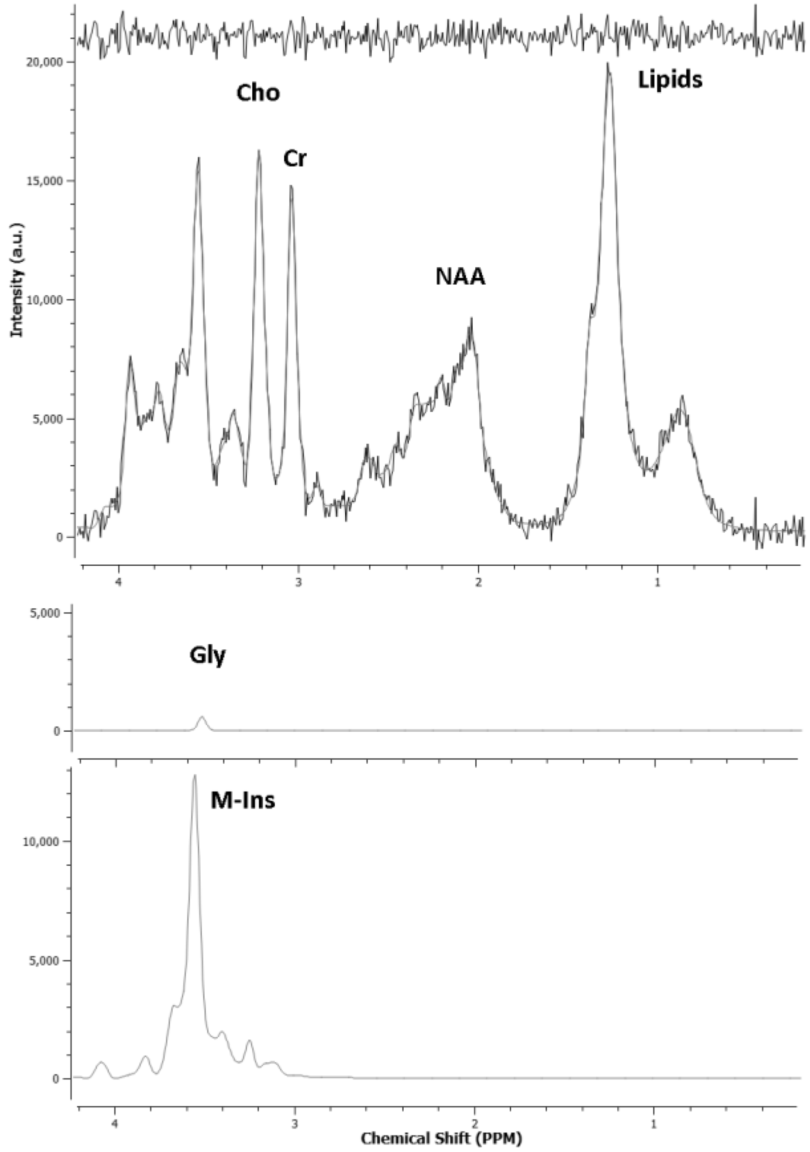

Supplementary Figure 1: An example ependymoma patient MRS spectrum with TARQUIN peak fits of Gly, M-Ins, baseline and fit risiduals shown. The ependymoma spectrum shows low Gly, high M-Ins and this patient survived within the 5 year follow-up period.

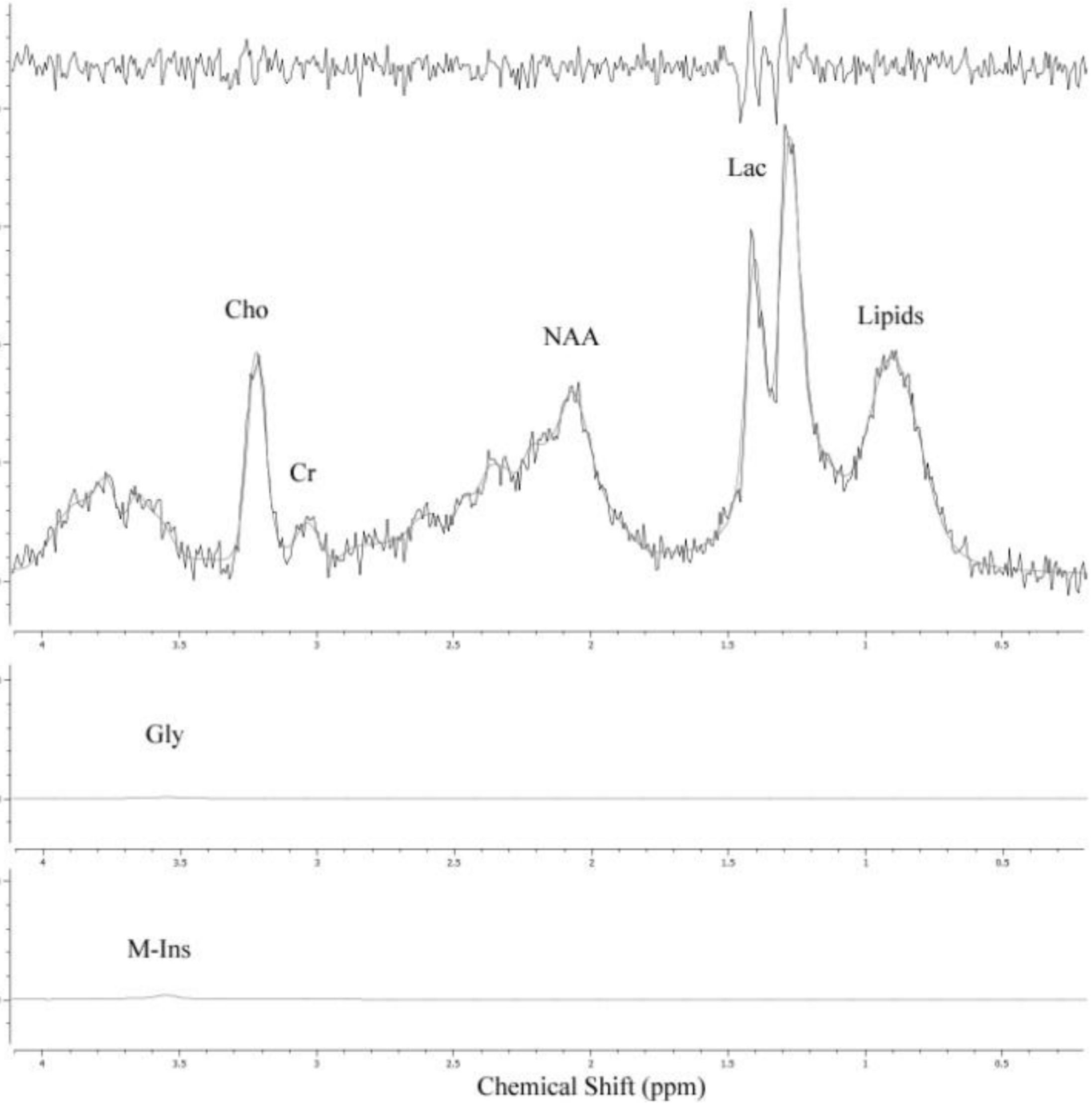

Supplementary Figure 2: An example pilocytic astrocytoma patient MRS spectrum with TARQUIN peak fits of Gly, M-Ins, baseline, and fit risiduals shown. The pilocytic astrocytoma spectrum shows low Gly, low M-Ins and this patient survived within the 5 year follow-up period.

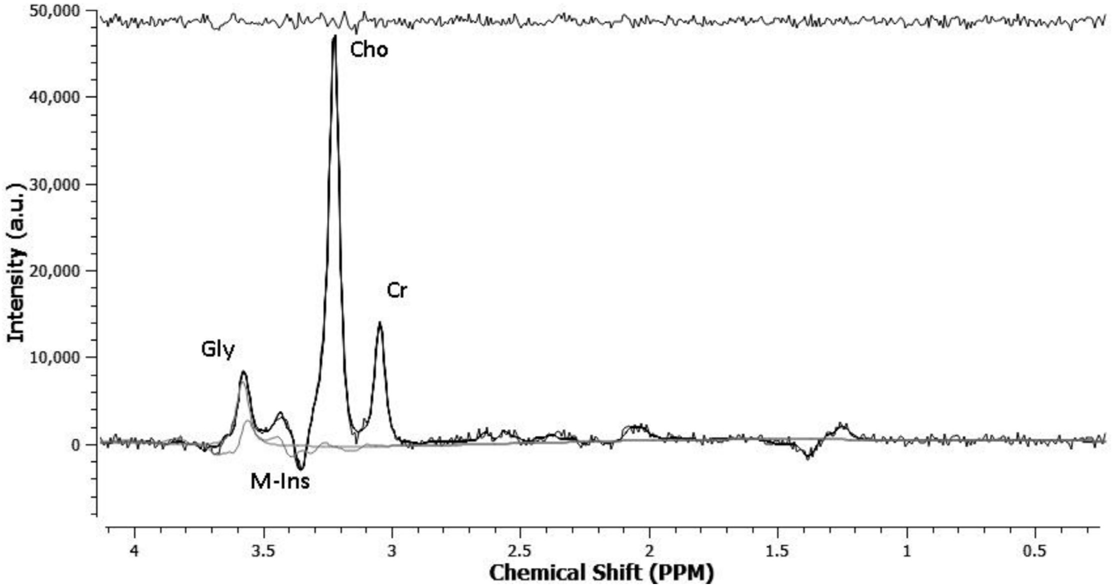

Supplementary Figure 3: An example of a medulloblastoma patient MRS long echo spectrum with TARQUIN peak fits of Gly, M-Ins, baseline, and fit risiduals shown.

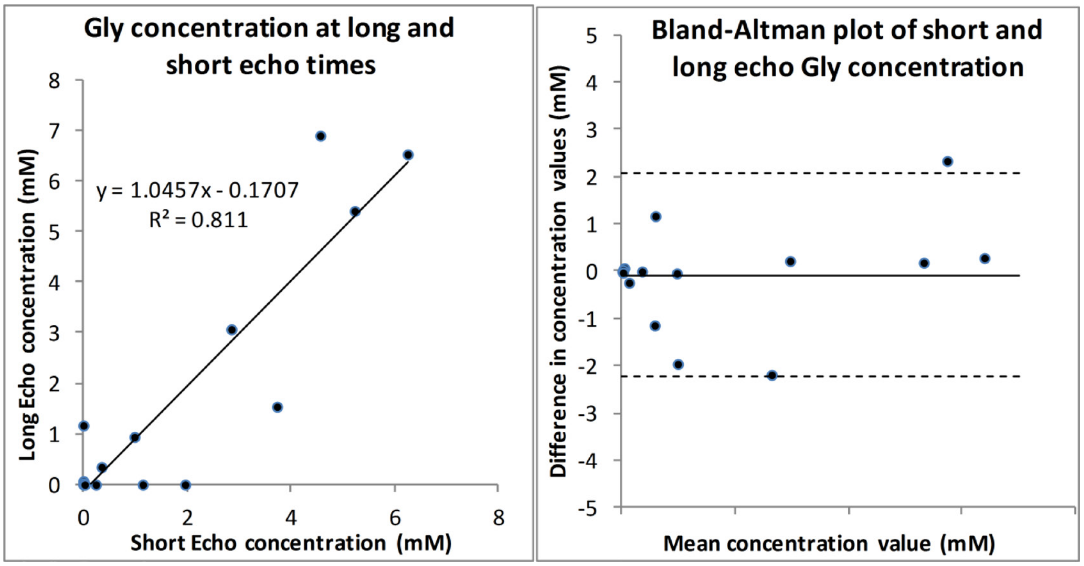

Supplementary Figure 4: A correlation curve (left) and a Bland-Altman plot (right) of Gly concentration in patients with both short and long echo MRS.
